# Efficacy and Safety of Chin Augmentation Using MaiLi-E, a Lidocaine-Containing Cross-Linked Sodium Hyaluronate Gel

**DOI:** 10.1007/s00266-025-04806-y

**Published:** 2025-04-21

**Authors:** Yun Xie, Yi Zhao, Dian Chen, Wei Lai, Zijian Gong, Hang Wang, Guo Li, Hongyi Zhao, Rongxin Ren, Jinping Ding, Jianling Shi, Yuanyuan Zhao, Nan Dai, Meng Yan, Fei Qin, Zhongxing Zhang, Qingfeng Li

**Affiliations:** 1 Shanghai Ninth People’s Hospital, Shanghai Jiaotong University School of Medicine, Shanghai, 200011 China; 2 Beijing Tsinghua Changgeng Hospital, Beijing, 102218 China; 3 The Third Affiliated Hospital of Sun Yat-sen University, Guangzhou, 510630 Guangdong China; 4 West China Hospital of Stomatology Sichuan University, Chengdu, 610041 Sichuan China; 5 Department of Plastic Surgery, Beijing Hospital, National Center of Gerontology, Institute of Geriatric Medicine, Chinese Academy of Medical Sciences, Beijing, 100730 People’s Republic of China; 6 Hangzhou Zhongmei HuaDong Pharmaceutical Co., Ltd., Hangzhou, 310020 China; 7 Sinclair Pharmaceuticals (Shanghai) Co., Ltd., Shanghai, 200003 China

**Keywords:** Randomized controlled trial, Lidocaine, Sodium hyaluronate gel, Injection, Chin augmentation

## Abstract

**Background:**

MaiLi-E is a lidocaine-containing cross-linked sodium hyaluronate gel for dermal filling. This prospective, multicenter, randomized, delayed-treatment controlled, evaluator-blinded clinical trial aims to evaluate the efficacy and safety of MaiLi-E for chin augmentation.

**Methods:**

Participants with mild-to-moderate–severe chin retrusion were enrolled and randomized (2:1) to receive MaiLi-E at study onset (MaiLi-E group) or six months later (control group). The primary efficacy endpoint was the response rate of chin retrusion improvement, defined as the percentage of participants whose China (Allergan) Chin Retrusion Scale score improved by ≥1 point from baseline, assessed by independent investigators at Month 6 post-injection for the MaiLi-E group and Month 6 post-randomization for the control group. Safety assessments included adverse events (AEs) and treatment-related AEs.

**Results:**

Between September 2022 and January 2023, 159 participants were enrolled (MaiLi-E group, *n* = 106; control group, *n* = 53). Response rates of chin retrusion improvement were 64.2% (95% CI 55.0–73.3%) in the MaiLi-E group and 20.8% (95% CI 9.8–31.7%) in the control group (*P* < 0.0001). Improvement rates of the Global Aesthetic Improvement Scale evaluated by participants were 91.5% (95% CI 86.2–96.8%) in the MaiLi-E group and 0.0% (95% CI 0.0–0.0%) in the control group. Response was maintained in most participants at Month 12 post-treatment. Participant satisfaction rates were 72.6% in the MaiLi-E and 74.5% in the control group. 64.8% of participants experienced AEs, and 5.0% experienced treatment-related AEs from the initial treatment to Month 6 after the last injection.

**Conclusion:**

MaiLi-E is effective and safe in correcting mild-to-moderate–severe chin retrusion. Evidence obtained from at least one properly designed randomized controlled trial.

**Level of Evidence I:**

This journal requires that authors assign a level of evidence to each article. For a full description of these Evidence-Based Medicine ratings, please refer to the Table of Contents or the online Instructions to Authors www.springer.com/00266.

**Supplementary Information:**

The online version contains supplementary material available at 10.1007/s00266-025-04806-y.

## Introduction

The chin contour is a key factor affecting facial profile attractiveness [1]. The structure of the chin includes skin, muscle, fat and bony structures, with the mandibular skeletal framework being the primary factor in determining the shape and contour of the chin. Correcting chin retrusion is necessary for enhancing facial esthetics. Chin contour can be altered through chin augmentation surgery, such as osteotomy, heterogeneous prosthesis implantation, autologous fat grafting and dermal filler injection [2].

Osteotomy-based chin augmentation, which permanently alters the structure of the mandible, is associated with an increased risk of complications such as infection, mental neuropraxia and chin hematoma, with a complication incidence rate of up to 19.7% [3, 4]. Alloplastic implants can achieve chin augmentation from a deep layer, but do not provide the benefits of tissue filling treatments. They are also associated with a complication rate of 15.7%, including bone resorption, malposition and nerve-related issues [3, 4]. Although autologous fat grafting is considered safe, it has limited efficacy on correcting chin height [5]. In contrast, chin hyaluronic acid (HA) injections are simple to perform and can effectively improve chin shape and contour without severe complications, making them a preferred choice among individuals seeking esthetic improvements [6].

HA is a naturally occurring macromolecule extensively present in the connective and epithelial tissues of mammals [7]. Renowned for its high hydrophilicity and exceptional water-binding capacity, HA undergoes a process of isochoric degradation. Its lack of species and tissue specificity further contributes to its outstanding biocompatibility [8]. Despite the presence of hyaluronidase in the human body, which reduces the natural half-life of HA to 1–2 days, cross-linking and modification techniques, either physical or chemical, can be employed to prolong its residence time in vivo [9].

MaiLi-E (Kylane, Swiss) incorporates OXIFREE^TM^ technology, which allows the preservation of the intrinsic properties of high molecular weight HA chains, providing optimized cohesivity and advanced rheological properties [10, 11]. This technology enhances the fillers’ projection capabilities and volumizing effect on facial skin tissues [10, 11], resulting in strong projection capabilities, pliability and a remarkable capacity for volumetric enhancement in facial esthetics [12]. MaiLi-E, with its higher projection capacity, may achieve the same volumizing effect as other HA fillers, but with a smaller injection volume, thereby offering an efficient and satisfactory treatment option for facial volume restoration [11].

However, the efficacy and safety of MaiLi-E for chin augmentation have not been formally assessed in China. Therefore, this clinical trial aims to evaluate the efficacy and safety of MaiLi-E for correcting chin retrusion.

## Methods

### Study Design and Participants

This is a prospective, multicenter, randomized, delayed-treatment controlled, evaluator-blinded clinical trial. Participants of Chinese origin with mild-to-moderate–severe chin retrusion were enrolled in five centers in China from September 2022 to January 2023. The study adhered to the Declaration of Helsinki, was approved by the ethics committees of each participating center and registered at chictr.org.cn (ChiCTR2200063817). Informed consent was obtained from all participants prior to enrollment.

The detailed inclusion and exclusion criteria are provided in the Supplemental Digital Content 5. Key inclusion criteria were: (1) adults aged 18 to 65 years (inclusive), of either sex; (2) willingness to receive chin filling treatment; and 3) mild-to-moderate–severe chin retrusion, defined as China (Allergan) Chin Retrusion Scale (CACRS) [13] score of 1–3 (Supplemental Digital Content 1), assessed by a blinded investigator. Key exclusion criteria were: (1) known allergic reactions to sodium hyaluronate acid products or any ingredients of the study product; (2) known allergic reactions to any local anesthetic agents (e.g., lidocaine or other amide anesthetics); (3) history of severe allergic reactions or multiple severe allergies; (4) abnormal coagulation functions (activated partial thromboplastin time >1.5 times the upper limit of normal) at screening, or treatment with any thrombolytic agent, anticoagulant or antiplatelet drug (e.g., warfarin and aspirin) within 2 weeks prior to enrollment; (5) previous use of permanent or semipermanent fillers (e.g., calcium hydroxyapatite, poly-L-lactic acid, polymethyl methacrylate, silicone, expanded polytetrafluoroethylene and polycaprolactone) or autologous fat in the chin; (6) moderate-to-severe chin retrusion, potentially accompanied by sleep-disordered breathing and requiring genioplasty; or (7) deemed unsuitable for the study by investigators.

### Intervention

MaiLi-E comprises HA at 24 mg/mL, phosphate buffer and lidocaine hydrochloride at 3 mg/mL. The total injection dose was limited to 4 mL at initial treatment and 2 mL for touch-ups across all treatment areas.

Participants were randomized in a 2:1 ratio to either receive MaiLi-E injection (MaiLi-E group) or undergo a 6-month control period, followed by delayed treatment with MaiLi-E injection (control group).

Participants in the MaiLi-E group received MaiLi-E injection on Day 0 post-randomization. If, at one month after the initial treatment, the injection investigators determined that the CACRS score improvement was less than 1 point and/or the esthetic effect was not optimal, a touch-up was administered with the participants’ consent.

Participants in the control group did not receive any injections on Day 0 post-randomization. After completing the efficacy and safety assessments at Month 6 post-randomization, those who met the treatment criteria entered the delayed treatment period and received MaiLi-E injections. Similarly, at one month after the initial treatment, if the injection investigators determined that the improvement in CACRS score was less than 1 point and/or the esthetic effect was not optimal, a touch-up was administered with the participants’ consent. Participants who did not meet the treatment criteria were withdrawn from the study.

### Visits

Participants in the MaiLi-E group were assessed for pain using the visual analogue scale (VAS) immediately and 30 minutes after the MaiLi-E injection. Participants in the control group returned to the center for efficacy and safety assessments at Months 1, 3 and 6 post-randomization. During the delayed treatment period, pain assessments were performed immediately and 30 minutes after the MaiLi-E injection. The efficacy, safety and participant satisfaction with MaiLi-E were evaluated at Months 1, 3, 6, 9 and 12 after the last injection.

### Endpoints

The primary efficacy endpoint was the response rate of chin retrusion improvement, evaluated by blinded independent investigators at Month 6 post-injection for the MaiLi-E group and at Month 6 post-randomization for the control group. The response rate was defined as the percentage of participants with a CACRS score improvement of ≥1 point from baseline.

The secondary efficacy endpoints were: (1) the response rate of chin retrusion improvement, assessed by blinded independent investigators, blinded investigators at each center and the injection investigators; (2) the improvement rate on the Global Aesthetic Improvement Scale (GAIS); (3) average pain levels, measured using the VAS; (4) participant satisfaction rate; and (5) change in chin volume.

The safety endpoints included: (1) the incidence and number of adverse events (AEs) and (2) the incidence and number of treatment-related AEs (TRAEs).

### Statistical Analysis

This study followed a superiority design. The sample size was calculated to achieve a power of 0.9 for detecting a difference in response rate of chin retrusion improvement, with 60% expected in the MaiLi-E group and 30% in the control group, using a one-sided significance level of α = 0.025. Consequently, the target enrollment was set at 159 participants, with 106 in the MaiLi-E group and 53 in the control group.

The full analysis set (FAS) included all randomized participants. The safety set (SS) included participants who received the trial product and underwent safety evaluation. Efficacy analysis was based on the FAS, and safety analysis was based on the SS.

The response rates of chin retrusion improvement and their 95% confidence interval (CI) in the MaiLi-E and control groups at Month 6 were calculated, along with the difference in effective rates between the groups with 95% CI. The Wald method was used to calculate the 95% CIs. For secondary efficacy endpoints, descriptive results were provided based on the data distribution characteristics. The quantitative indicators were compared between groups using Student’s t-test or the Wilcoxon rank-sum test, while categorical indicators were tested using the Chi-squared test or exact probability test. The differences in rates and means were calculated, along with their 95% CIs. The incidence, number and severity of AEs, TRAEs, device defects and local reactions at the injection site were recorded throughout the entire study period. All statistical analyses were conducted using SAS 9.4 (SAS Institute, Cary, NC, USA). Two-sided P-values <0.05 were considered statistically significant.

## Results

### Baseline Characteristics and Treatments

**Fig. 1:**
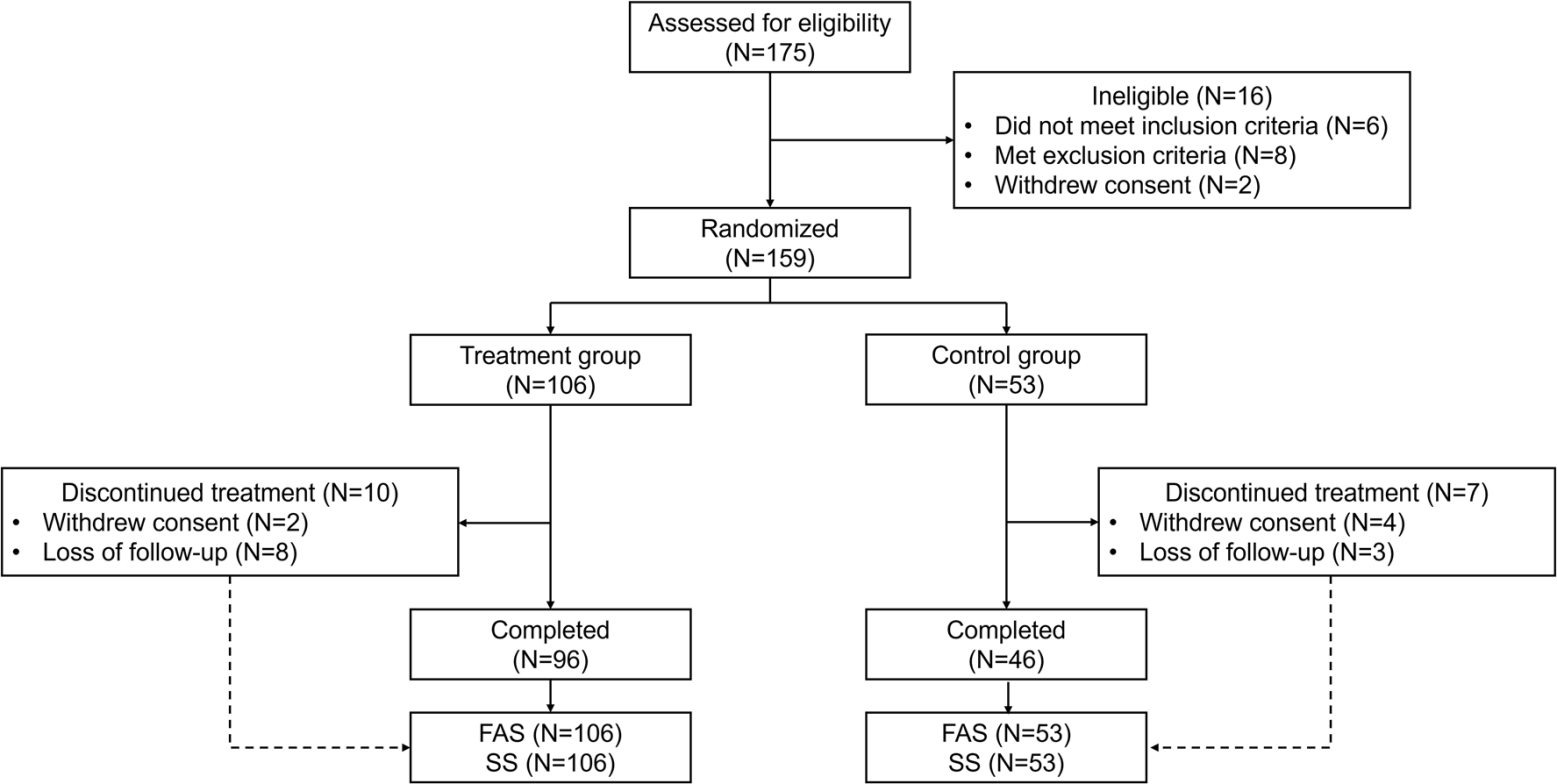
Study flowchart

A total of 175 participants were screened, with 16 (9.1%) excluded due to screen failure, resulting in 159 enrolled participants (106 in the MaiLi-E group and 53 in the control group). All participants (100%) were included in the FAS and SS (Fig. 1).

The baseline characteristics of the participants are presented in Table 1, and the injection status is detailed in Table 2. All participants in the MaiLi-E group received the initial treatment, with 39 (36.8%) also receiving a touch-up one month later. In the control group, 51 (96.2%) participants entered the delayed treatment period and received the initial treatment at Month 6 post-randomization, and 28 (52.8%) also received a touch-up one month after their initial treatment. The average total dose was 2.31 ± 1.05 mL for the MaiLi-E group and 2.83 ± 1.07 mL for the control group. For the MaiLi-E group, the average total doses for initial treatment and touch-up were 1.90 ± 0.75 and 1.11 ± 0.49 mL, respectively, comparable to 2.13 ± 0.74 mL and 1.27 ± 0.47 mL, respectively, for the control group.

**Table 1:** Baseline characteristics of the participants (FAS)

Variables	MaiLi-E group (*n* = 106)	Control group (*n* = 53)	All (*n* = 159)
Age (years)	34.0±8.96	34.5±7.88	34.2±8.60
Gender			
Female	11 (10.4)	6 (11.3)	17 (10.7)
Male	95 (89.6)	47 (88.7)	142 (89.3)
Ethnicity			
Han Chinese	100 (94.3)	48 (90.6)	148 (93.1)
Others^a^	6 (5.7)	5 (9.4)	11 (6.9)
Participants with plastic surgery history	19 (17.9)	9 (17.0)	28 (17.6)
Type of plastic surgery			
Sodium hyaluronate skin filler	7 (6.6)	2 (3.8)	9 (5.7)
Botulin	7 (6.6)	1 (1.9)	8 (5.0)
Cosmetic facial procedures	4 (3.8)	3 (5.7)	7 (4.4)
Laser or chemical stripping	2 (1.9)	2 (3.8)	4 (2.5)
Mesotherapy	1 (0.9)	1 (1.9)	2 (1.3)
Lower eyelid blepharoplasty	1 (0.9)	0	1 (0.6)
Double eyelid operation	1 (0.9)	0	1 (0.6)
Unknown	0	1 (1.9)	1 (0.6)
Involved areas			
Chin	5 (4.7)	2 (3.8)	7 (4.4)
Lips	4 (3.8)	1 (1.9)	5 (3.1)
Entire face	4 (3.8)	1 (1.9)	5 (3.1)
Orbital areas	2 (1.9)	3 (5.7)	5 (3.1)
Forehead	3 (2.8)	1 (1.9)	4 (2.5)
Lower face	2 (1.9)	0	2 (1.3)
Cheeks	1 (0.9)	0	1 (0.6)
Cheeks and forehead	0	1 (1.9)	1 (0.6)
Infraorbital region	1 (0.9)	0	1 (0.6)
Midface	0	1 (1.9)	1 (0.6)
Parotideomasseteric region	1 (0.9)	0	1 (0.6)
Calves	1 (0.9)	0	1 (0.6)
Axillary	1 (0.9)	0	1 (0.6)

All data are shown as mean ± standard deviation or *n* (%)

^a^Hui, Mongolian, Miao, Yi and Zhuang

**Table 2:** Injection volumes

	MaiLi-E group (*n* = 106)	Control group (*n* = 53)
Initial treatment		
Received injection, *n* (%)	106 (100)	51 (96.2)
Volume (mL), mean ± SD	1.90 ± 0.75	2.13 ± 0.74
Touch-up		
Received injection, *n* (%)	39 (36.8)	28 (52.8)
Volume (mL), mean ± SD	1.11 ± 0.49	1.27 ± 0.47
Total		
Received injection, *n* (%)	106 (100.0)	51 (96.2)
Volume (mL), mean ± SD	2.31 ± 1.05	2.83 ± 1.07

### Primary Efficacy Endpoint

The response rate of chin retrusion improvement, as evaluated by blinded independent investigators, was 64.2% (95% CI 55.0–73.3%) in the MaiLi-E group at Month 6 after the last injection and 20.8% (95% CI 9.8–31.7%) in the control group at Month 6 after randomization. The difference in response rates between the groups was 43.4% (95% CI 29.2–57.6%; *P* < 0.0001), indicating a significantly higher response rate in the MaiLi-E group compared to the control group (Table 3). Representative photographs of subjects before and after treatment are shown in Fig. 3.

**Table 3:** Efficacy endpoint at Month 6 (full analysis set)

Evaluator	Endpoint	MaiLi-E group (*n* = 106)	Control group (*n* = 53)	Difference	*P*
Blinded independent investigators	Response rate of chin retrusion improvement ^a^, *n* (%)	68 (64.2)	11 (20.8)	43.4	<0.0001
	95% CI	(55.0, 73.3)	(9.8, 31.7)	(29.2, 57.6)	
Blinded investigators in the center	Response rate of chin retrusion improvement, *n* (%)	72 (67.9)	3 (5.7)	62.3	<0.0001
	95% CI	(59.0, 76.8)	(0, 11.9)	(51.4, 73.1)	
Injection investigators	Response rate of chin retrusion improvement, *n* (%)	82 (77.4)	1 (1.9)	75.5	<0.0001
	95% CI	(69.4, 85.3)	(0, 5.5)	(66.7, 84.2)	
Injection investigators	Improvement rate of GAIS score, *n* (%)	100 (94.3)	2 (3.8)	90.6	<0.0001
	95% CI	(89.9, 98.7)	(0, 8.9)	(83.8, 97.3)	
Participants	Improvement rate of GAIS score, *n* (%)	97 (91.5)	0	91.5	<0.0001
	95% CI	(86.2, 96.8)	0 (0, 0)	86.2, 96.8	
–	Change in chin volume (mL)				
	*n* (%)	100 (94.3)	52 (98.1)		
	Mean ± SD	2.968±1.9385	0.088±1.5688		<0.0001

*CI*: Confidence interval; *GAIS*: Global Aesthetic Improvement Scale

^a^Missing data was filled in using the WOCF method. Missing values at other endpoints were treated as no response

### Secondary Efficacy Endpoints

**Fig. 2:**
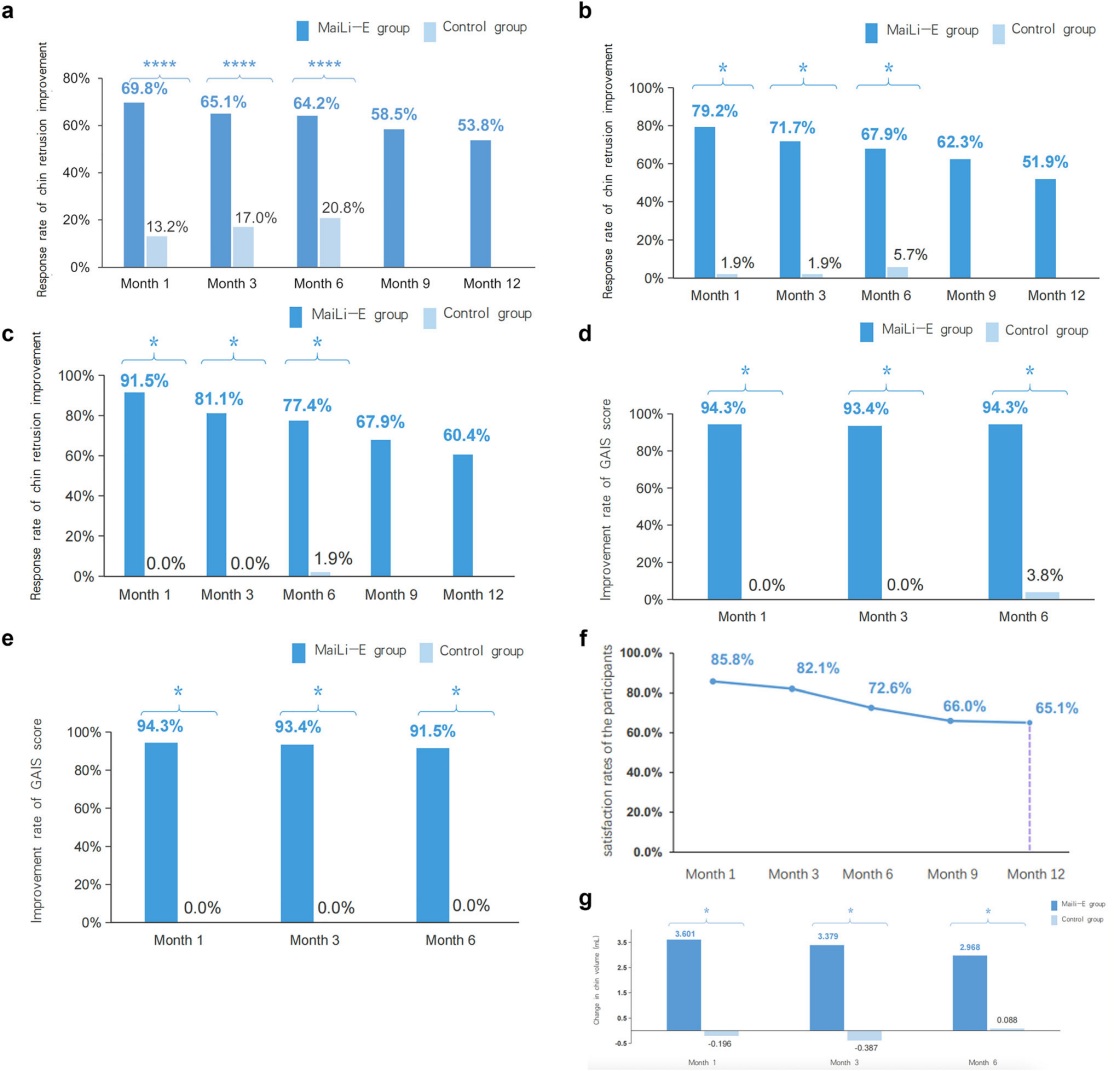
Efficacy endpoints (full analysis set). **a** Response rate of chin retrusion improvement evaluated by blinded independent investigators. **b** Response rate of chin retrusion evaluated by blinded investigators in each center. **c** Response rate of chin retrusion evaluated by injection investigators. **d** Improvement rate of GAIS score evaluated by injection investigators. **e** Improvement rate of GAIS score evaluated by participants. **f** Participant satisfaction. **g** Changes in chin volume in the MaiLi-E group and in the control group. *P < 0.0001

The response rate of chin retrusion was significantly higher in the MaiLi-E group compared to the control group at Months 1 and 3 after treatment, as evaluated by independent blinded investigators (*P* < 0.05) (Fig. 2a). At Month 12 after the last treatment, the response rate of chin retrusion improvement in the MaiLi-E group was 53.8% (95% CI 44.3–63.3%), as evaluated by independent blinded investigators. Evaluations by blinded investigators at each center showed a response rate of 67.9% (95% CI 59.0–76.8%) in the MaiLi-E group and 5.7% (95% CI 0.0–11.9%) in the control group at Month 6 after randomization. The response rate of chin retrusion improvement, as evaluated by the injection investigators, was 77.4% (95% CI 69.4–85.3%) in the MaiLi-E group at Month 6 after the last treatment and 1.9% (95% CI 0.0–5.5%) in the control group at Month 6 after randomization (Table 3). Similar results were observed at Months 1 and 3 (all *P* < 0.05) (Fig. 2b–c).

The improvement rates of the GAIS score, as evaluated by the injection investigators, were 94.3% (95% CI 89.9–98.7%) in the MaiLi-E group at Month 6 after the last treatment and 3.8% (95% CI 0.0–8.9%) in the control group at Month 6 after randomization. Evaluations by the participants showed improvement rates of 91.5% (95% CI 86.2–96.8%) in the MaiLi-E group and 0.0% (95% CI 0.0–0.0%) in the control group (Table 3). Similar results were observed at Months 1 and 3 (*P* < 0.05) (Fig. 2d–e). At Month 12, after the last treatment for the MaiLi-E group, the improvement rates of the GAIS score were 86.8% (95% CI 80.3–93.2%) and 80.2% (95% CI 72.6–87.8%), as assessed by the injection investigators and the participants, respectively.

The degree of pain at the injection site is detailed in Supplemental Digital Content 2. Immediately after the initial treatment, the VAS pain scores of the participants in the MaiLi-E and control groups were 2.3 ± 1.86 and 2.0 ± 1.66, respectively. At 30 minutes after the initial treatment, the VAS pain scores were 1.1 ± 1.52 and 1.1 ± 1.20, respectively. Similar VAS pain scores were observed following the touch-up treatment.

The satisfaction rates of the participants at Month 6 after the last injection in the MaiLi-E and control group were 72.6% and 74.5%, respectively (Supplemental Digital Content 3 and Fig. 2f).

The changes in chin volume from baseline in the MaiLi-E group at Months 1, 3 and 6 after the last treatment were 3.60 ± 1.80 mL, 3.38 ± 1.95 mL and 2.97 ± 1.94 mL, respectively. For the control group, the changes in chin volume from baseline at Months 1, 3 and 6 after randomization were − 0.20 ± 1.11 mL, − 0.39 ± 1.02 mL and 0.09 ± 1.57 mL, respectively (Fig. 2g).

**Fig. 3:**
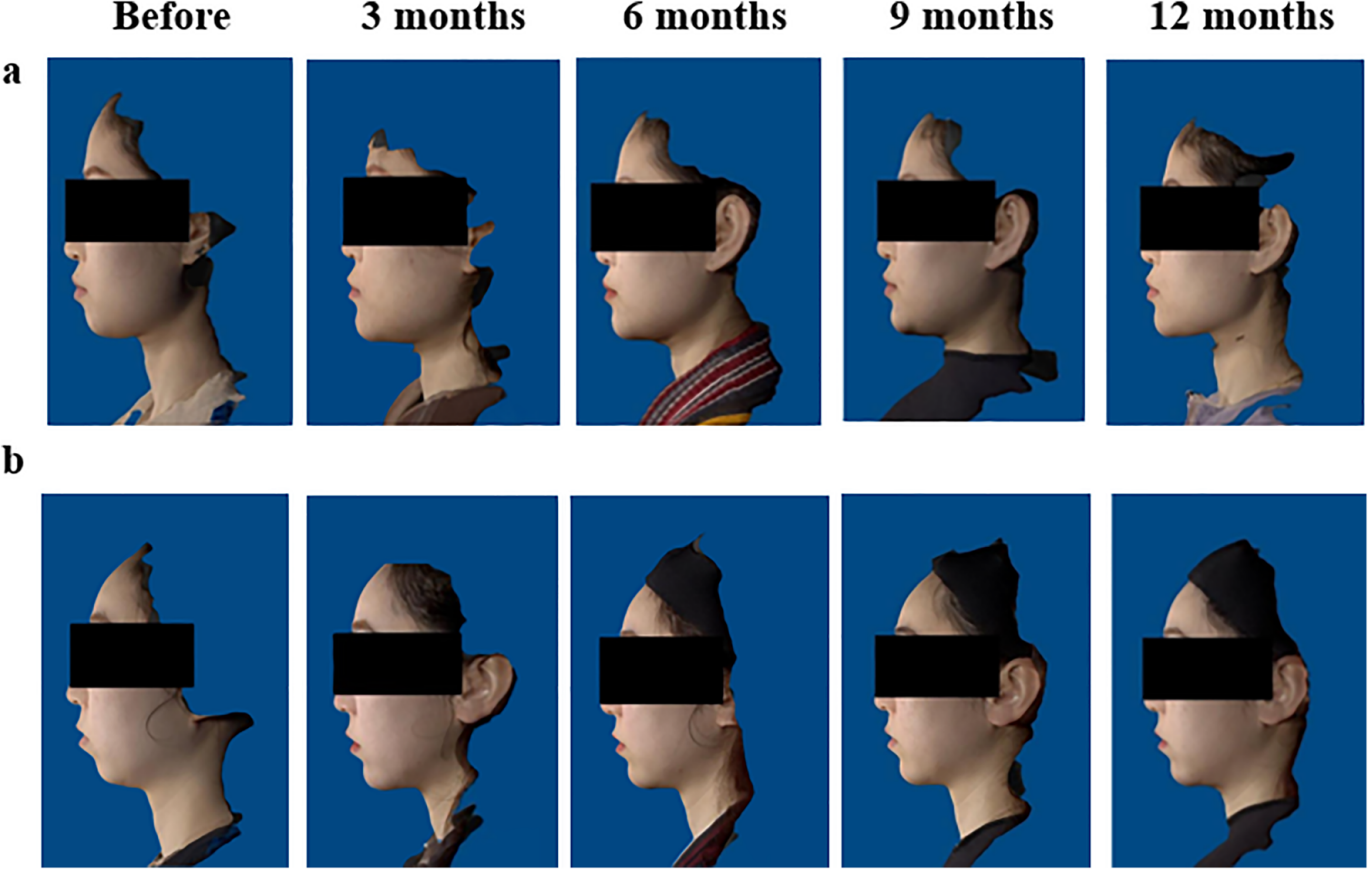
Representative photographs of a participant before treatment and at 3, 6, 9 and 12 months after treatment (left to right). **a** A 29-year-old woman who received an initial treatment of 2.0 mL MaiLi-E without touch-up treatment. **b** A 28-year-old woman who received an initial treatment of 3.0 mL MaiLi-E without touch-up treatment

Figure 3 shows photographs of the same participants taken before treatment and at 3, 6, 9 and 12 months after treatment.

### Safety

From the initial treatment to six months after the last injection, 64.8% of the participants experienced AEs, and 5.0% experienced TRAEs. In the MaiLi-E group, 66.0% (70/106) of the participants experienced AEs from the initial treatment to six months after the last injection. In the control group, 62.3% (33/53) of the participants experienced AEs from randomization to before the initial treatment. All AEs in both groups were mild or moderate. Since this study was conducted during the COVID-19 pandemic in China, most AEs were COVID-19 infections (71.4% in the MaiLi-E group and 72.7% in the control group). A total of 7.5% (8/106) of the participants in the MaiLi-E group experienced TRAEs. The most common TRAEs in the MaiLi-E group included injection site induration (4.7%), injection site nodule (2.8%), injection site pain (1.9%) and injection site lump (1.9%). Two participants (1.9%) in the MaiLi-E group experienced serious adverse events deemed unrelated to MaiLi-E. No participant experienced AEs leading to withdrawal from the study or death (Table 4).

**Table 4:** Summary of adverse events (safety set)

	MaiLi-E group (*n* = 106)	Control group (*n* = 53)
AE	70 (66.0)	33 (62.3)
TRAE	8 (7.5)	0
SAE	1 (0.9)	0
SAE related to treatment	0	0
Injection site reaction-related events	9 (8.5)	0
AEs leading to withdrawal	0	0
AEs leading to death	0	0
Summary of TRAE by preferred terms		
Injection site induration	5 (4.7)	0
Injection site nodule	3 (2.8)	0
Injection site pain	2 (1.9)	0
Injection site lump	2 (1.9)	0
Injection site swelling	1 (0.9)	0
Injection site bleeding	1 (0.9)	0
Injection site hematoma	1 (0.9)	0
Injection site hypoesthesia	0	0

All are shown as *n* (%)

*AE*: Adverse event; *TRAE*: treatment-related adverse event; *SAE*: serious adverse event

Stage 1 of the MaiLi-E group: from the initial treatment to Month 6 after the last injection. Stage 1 of the control group: from randomization to before the initial treatment

## Discussion

MaiLi-E is a lidocaine-containing cross-linked sodium hyaluronate gel for dermal filling. This randomized controlled trial evaluated the efficacy and safety of MaiLi-E for chin augmentation. The results demonstrated that the response rate of chin retrusion improvement, as evaluated by blinded independent investigators, was significantly higher in the MaiLi-E group after the last treatment compared to the control group at Month 6 post-randomization. Similar improvements were observed for the response rate of chin retrusion, improvement rate of GAIS score and chin volume change at Months 1, 3 and 6 after the last treatment, all of which were superior to the control group. The treatment effect was maintained for 12 months after the last treatment in the MaiLi-E group according to the CACRS score, and the overall cosmetic effect remained favorable for most participants according to the GAIS score. In addition, the reported pain was mild.

The efficacy evaluation results of this study support MaiLi-E as an effective agent for chin augmentation. In this study, the response rate of chin retrusion improvement, evaluated by independent blinded investigators, was 64.2% (95% CI 55.0–73.3%) in the MaiLi-E group at Month 6 after the last treatment, significantly higher than the 20.8% (95% CI 9.8–31.7%) observed in the control group at Month 6 after randomization. The difference in rates between the groups was 43.4% (95% CI 29.2–57.6%). The efficacy of MaiLi-E for chin augmentation appears to be comparable to other cross-linked HA gels. Marcus et al. [14] reported that Restylane Defyne, a hyaluronic acid filler, markedly improved the response rate of chin retrusion improvement according to the Galderma Chin Retrusion Scale, as assessed by blinded evaluators, at 24 weeks (85.7 vs. 6.7%). Similar results were observed in Chinese participants (81.1 vs. 5.4%) at Month 6 following injection [15]. A study of VYC-20L, another lidocaine-containing HA filler, evaluated the response rate of chin retrusion improvement using the Allergan Chin Retrusion Scale and reported that 56.3% of participants achieved improvement of chin retrusion compared to 27.5% in the control group [16]. Regarding GAIS scores, the improvement rates in the MaiLi-E group at Month 6 after the last treatment were 94.3% and 91.5%, as assessed by the injection investigators and the participants, respectively. These rates were maintained to Month 12 post-treatment, with improvement rates of 86.8% and 80.2%, as assessed by the injection investigators and the participants, respectively. These findings are comparable to other studies, where improvement rates of the GAIS score ranged from 83.5 to 97.0% by injection investigators and 77.2 to 84.8% by participants at Month 12 after the last treatment [14, 15, 17]. These results collectively indicate that MaiLi-E demonstrates satisfactory efficacy in chin augmentation, with outcomes comparable to those of analogous products. The augmentation effect persisted in the majority of participants, with favorable cosmetic outcomes sustained up to Month 12 after the final injection. Nevertheless, caution is warranted when comparing the findings of this study with those of other studies due to variations in baseline characteristics across study populations and minor discrepancies in evaluation scales. A literature search comparing the rheological parameters of MaiLi-E with other HA filling products suggests advantages of its elastic modulus (G’) and viscous modulus (G’’) over other products (Supplemental Digital Content 4). However, as the data are from different studies with varying experimental conditions [11, 18–20], they should be interpreted with caution and confirmed in future research. MaiLi-E required 1.90 ± 0.75 mL for the initial treatment and 1.11 ± 0.49 mL for touch-up treatment, lower than the 2.61 ± 0.957 mL and 1.35 ± 0.620 mL for Restylane Defyne, and slightly less than the 1.9 ± 0.6 mL and 1.4 ± 0.8 mL for VYC-20L [14, 16]. Additionally, only 36.8% of participants needed a touch-up treatment with MaiLi-E, significantly lower than the 73.6% for Restylane Defyne and 51.4% for VYC-20L [14, 16]. These findings suggest that MaiLi-E, with its enhanced projection capacity, could achieve comparable volumizing effects to other HA fillers with a smaller injection volume.

In the present study, only 7.5% of participants in the MaiLi-E group experienced TRAEs, comparable to rates observed in previous studies: 8% with VYC-20L [16], 30% with VYC-25L [17] and 14% with Restylane Defyne [14]. The most common TRAEs of MaiLi-E were injection site induration, injection site nodule, injection site pain and injection site lump, with no new safety signals identified compared to other products [14–17]. Of note, this trial was conducted during the COVID-19 pandemic, and while COVID-19 infection was the most common AE, it did not impact trial participation. The only two serious adverse events were moderate hemorrhoids (definitely unrelated to the study treatment) and moderate chest discomfort (potentially unrelated to the study treatment). No participants withdrew from the study due to AEs, and there were no deaths. These results highlight the good safety of MaiLi-E for chin augmentation.

A strength of the present study was its randomized controlled design with blind assessment, which minimized bias and controlled confounding factors, enhancing the reliability of the results. Nevertheless, this study had limitations. The trial used a blank control design due to the absence of approved similar products in the Chinese market at the time, which prevented direct efficacy comparisons with other products. Without a positive control group, the relative advantages and disadvantages of MaiLi-E cannot be clearly established and require further investigation. In addition, the follow-up was limited to one year. Future studies should extend follow-up to determine the long-term efficacy and safety of MaiLi-E.

## Conclusion

In conclusion, MaiLi-E injection is effective and safe for augmentation of the chin region, effectively correcting mild-to-moderate–severe chin retrusion. The majority of participants maintained the augmentation response at Month 12 after the last MaiLi-E injection.

## Supplementary Information

Below is the link to the electronic supplementary material.

Supplementary file1 (DOCX 18 KB)

Supplementary file2 (DOCX 16 KB)

Supplementary file3 (DOCX 16 KB)

Supplementary file4 (DOCX 16 KB)

Supplementary file5 (DOCX 17 KB)
