# Supplementary material for: Efficacy and Safety of Chin Augmentation Using MaiLi-E, a Lidocaine-Containing Cross-Linked Sodium Hyaluronate Gel
Source: Aesthetic Plast Surg. 2025 Apr 21;49(11):3159–69. doi: 10.1007/s00266-025-04806-y (PMC12222339; doi:10.1007/s00266-025-04806-y)
Supplement: Supplementary file 1 — Supplementary file1 (DOCX 18 KB) [file 266_2025_4806_MOESM1_ESM.docx]

**Inclusion criteria**

To participate in this clinical trial, individuals had to meet the following criteria:

(1) Aged 18 to 65 years old (inclusive), male or female.

(2) Willingness to receive chin-filling treatment.

(3) With mild to moderate-severe chin retrusion (i.e., CACRS score of 1–3) as assessed by a blinded investigator on site.

(4) Voluntarily sign the informed consent form, understand and accept the duration of the study, and be able and willing to comply with all requirements, including scheduled treatment, follow-up, and other study procedures.

**Exclusion criteria**

Participants meeting any of the following exclusion criteria are ineligible for enrollment in the study.

(1) Known allergic reactions to sodium hyaluronate acid products or any of the ingredients contained in this product.

(2) Known allergic reactions to any local anesthetic agents (e.g., lidocaine or other amide anesthetics).

(3) Known history of severe allergic reactions and multiple severe allergies.

(4) Abnormal coagulation mechanism (activated partial thromboplastin time >1.5 times the upper limit of normal) at screening, or having received any thrombolytic agent, anticoagulant, or antiplatelet drug (e.g., warfarin, aspirin) within 2 weeks prior to enrollment.

(5) Having received any permanent or semi-permanent fillers in the chin, e.g., calcium hydroxyapatite, poly-Llactic acid, polymethyl methacrylate, silicone, expanded polytetrafluoroethylene and polycaprolactone, autologous fat, etc.

(6) Having received other temporary fillers, such as sodium hyaluronate (including hydro lifting) or collagen injection in the chin and/or lips, facial lift (thread lift) within 12 months before enrollment.

(7) Having received any medical and aesthetic treatment (e.g., botulinum toxin injection, radiofrequency therapy, ultrasound therapy, laser or chemical peels, dermabrasion, facial lift including thread lift) in the lower face within 6 months before enrollment.

(8) Previous chin surgery that might affect the evaluation of efficacy as judged by the investigator.

(9) Presence of tattoos, piercings, significant facial hair (e.g., whiskers), scars, deformities, unhealed wounds, active skin diseases or inflammation or infections (e.g., herpes, acne, eczema, dermatitis, psoriasis, and herpes zoster), abscess, active or persistent oral infection, cancers or precancerous lesions, etc. in the lower face that may affect the efficacy evaluation or increase treatment-related risk.

(10) Active autoimmune diseases or medical history (e.g., systemic lupus erythematosus, rheumatoid arthritis, scleroderma, dermatomyositis, etc.) and with porphyria.

(11) Tendency to form keloids, hypertrophic scars, or any other healing disorders.

(12) Having received chemotherapy, immunosuppressants, immunomodulatory therapy (e.g., monoclonal antibodies), or systemic corticosteroids (except inhaled corticosteroids) within 3 months before enrollment.

(13) Severe dysfunction of vital organs (brain, heart, lung, liver, kidney, etc.) as judged by the investigator; individuals with serious cardiovascular and cerebrovascular diseases, serious lung diseases, liver function (alanine aminotransferase and aspartate aminotransferase >2 times the upper limit of normal, renal function (Cr) > 1.5 times the upper limit of normal, and uncontrolled diabetes.

(14) Moderate to severe chin retrusion, potentially accompanied by sleep-disordered breathing and requiring genioplasty.

(15) Plans to undergo any procedures or surgical treatments affecting the lower facial contour, such as face filling, wrinkle correction or surgery, tooth extraction, orthodontic treatment, dental implant, or denture fitting during the study.

(16) Participation in other clinical trials within 3 months before enrollment in this study or current participation in other clinical trials.

(17) Pregnant or lactating women.

(18) Women of childbearing potential who plan to become pregnant or do not use effective contraceptive measures during the study; female participants of childbearing potential should have a negative pregnancy test before the initial treatment; male participants who plan to have a child or are unwilling to use appropriate contraceptive measures during the study.

(19) Diagnosis of mental illness, incapable of autonomous behavior, or current episodes of anxiety or depression.

(20) Any condition that the investigator deems inappropriate for participation in the clinical study.
