# Supplementary material for: Efficacy and Safety of Chin Augmentation Using MaiLi-E, a Lidocaine-Containing Cross-Linked Sodium Hyaluronate Gel
Source: Aesthetic Plast Surg. 2025 Apr 21;49(11):3159–69. doi: 10.1007/s00266-025-04806-y (PMC12222339; doi:10.1007/s00266-025-04806-y)
Supplement: Supplementary file 2 — Supplementary file2 (DOCX 16 KB) [file 266_2025_4806_MOESM2_ESM.docx]

**Table S1.** Descriptors for the China (Allergan) Chin Retrusion Scale

| Score | Grade | Descriptor |
| --- | --- | --- |
| 0 | None | No chin retrusion; defined chin midpoint at or in front of the labrale inferius line |
| 1 | Minimal | Minimal chin retrusion; defined chin midpoint between the supramentale line and the labrale inferius line |
| 2 | Moderate | Moderate chin retrusion; defined chin midpoint at the supramentale line |
| 3 | Moderate-Severe | Moderate-severe chin retrusion; defined chin midpoint between the supramentale line and the cheilion vertical line |
| 4 | Severe | Severe chin retrusion; defined chin midpoint at or behind the cheilion vertical line |
