# Supplementary material for: Efficacy and Safety of Chin Augmentation Using MaiLi-E, a Lidocaine-Containing Cross-Linked Sodium Hyaluronate Gel
Source: Aesthetic Plast Surg. 2025 Apr 21;49(11):3159–69. doi: 10.1007/s00266-025-04806-y (PMC12222339; doi:10.1007/s00266-025-04806-y)
Supplement: Supplementary file 3 — Supplementary file3 (DOCX 16 KB) [file 266_2025_4806_MOESM3_ESM.docx]

**Table S2.** VAS score (FAS)

| Evaluation timepoint | MaiLi-E group | Control group |
| --- | --- | --- |
| Immediately after the initial treatment | 2.3±1.86 | 2.0±1.66 |
| 30±3 min after the initial treatment | 1.1±1.52 | 1.1±1.20 |
| Immediately after the touch-up | 1.5±1.70 | 1.6±1.59 |
| 30±3 min after the touch-up | 1.1±1.41 | 1.1±1.39 |

FAS, full analysis set; VAS, visual analogue scale.
