# Supplementary material for: Efficacy and Safety of Chin Augmentation Using MaiLi-E, a Lidocaine-Containing Cross-Linked Sodium Hyaluronate Gel
Source: Aesthetic Plast Surg. 2025 Apr 21;49(11):3159–69. doi: 10.1007/s00266-025-04806-y (PMC12222339; doi:10.1007/s00266-025-04806-y)
Supplement: Supplementary file 4 — Supplementary file4 (DOCX 16 KB) [file 266_2025_4806_MOESM4_ESM.docx]

**Table S3.** The satisfaction rate of the participants at Month 6 after the last injection (FAS)

| Endpoint | MaiLi-E group  (n=106) | Control group  (n=51) |
| --- | --- | --- |
| Satisfaction rate at month 6 after the last injection | 77 (72.6) | 38 (74.5) |
