# Supplementary material for: Efficacy and Safety of Chin Augmentation Using MaiLi-E, a Lidocaine-Containing Cross-Linked Sodium Hyaluronate Gel
Source: Aesthetic Plast Surg. 2025 Apr 21;49(11):3159–69. doi: 10.1007/s00266-025-04806-y (PMC12222339; doi:10.1007/s00266-025-04806-y)
Supplement: Supplementary file 5 — Supplementary file5 (DOCX 17 KB) [file 266_2025_4806_MOESM5_ESM.docx]

**Table S4.** Rheological parameters of MaiLi-E and other hyaluronic acid fillers

| Product name | Cross-linking technology | HA content (mg/mL) | G’ (Pa) | G’’ (Pa) | Tan δ |
| --- | --- | --- | --- | --- | --- |
| MaiLi-E | Oxifree^TM^ | 24 | 244.22 ± 8.18 | 45 ± 2.31 | 0.1-0.2 |
| HA hydrogel 24 mg/mL | SXT | 24 | 82.34 ± 3.72 | 31.92 ± 1.49 | 0.39 ± 0.02 |
| Juvederm Voluma^*^ | Vycross^TM^ | 20 | 318 ± 3 | 41 | 0.103 |
| Restylane Volyme | XpresHAn | 20 | 239 | 50 | 0.209 |
| Restylane  Lyft | NASHA^TM^ | 20 | 977 | 198 | 0.203 |
| TEOSYAL RHA 4 | Preserved Network | 23 | 346 | 62 | 0.179 |

The G’ and G’’ of MaiLi-E and HA hydrogel 24 mg/mL were tested the at the oscillation frequency of 1 Hz; The G’ and G’’ of Restylane Volyme, Restylane Lyft and TEOSYAL RHA 4 were tested the at the oscillation frequency of 5 Hz.

^*^ The G' and G'' of VYC-20L were tested using oscillatory frequency sweeps at 1 Hz and 5 Hz, respectively.
